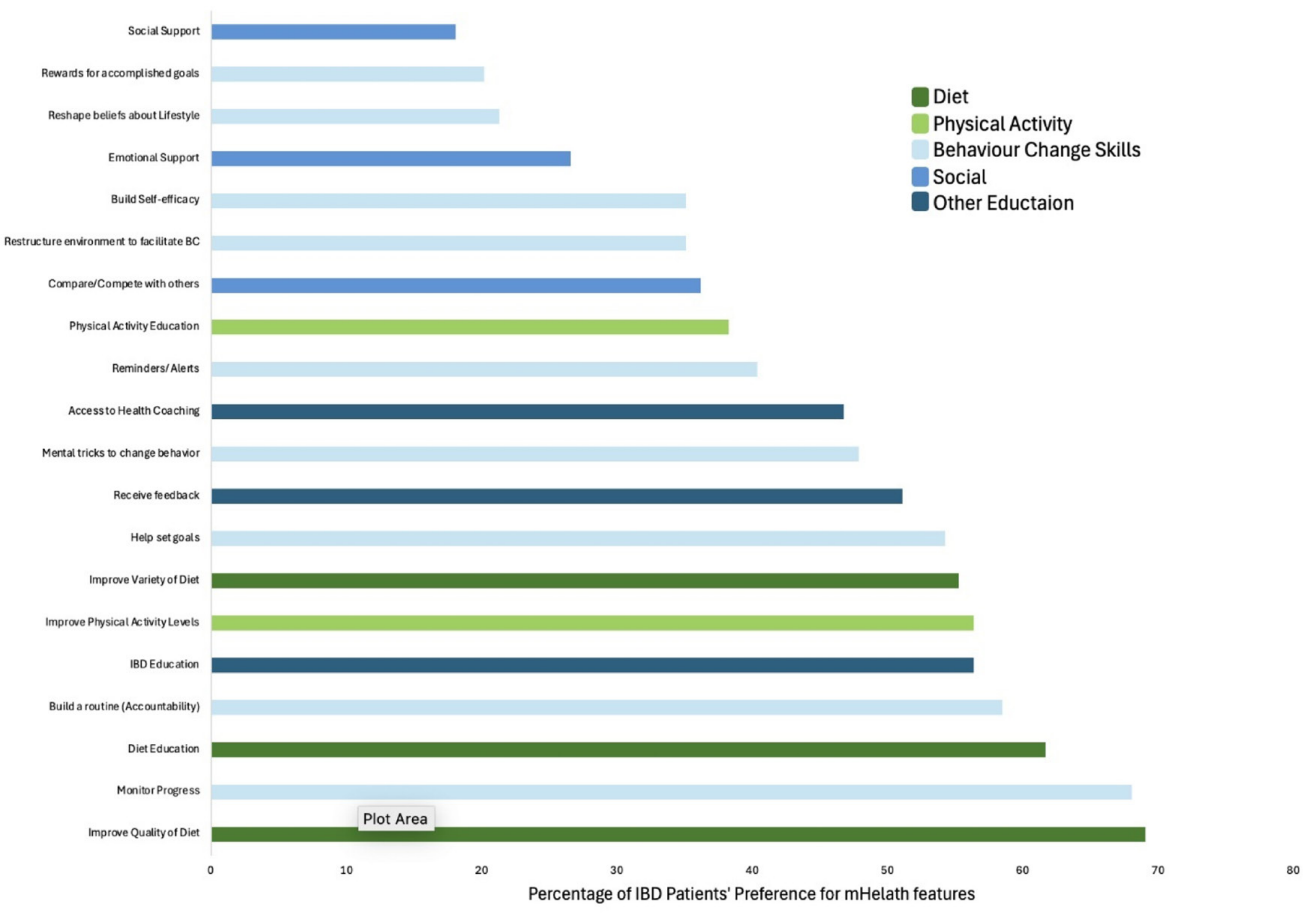# Poster Session I - A126HEALTH BEHAVIOUR CHANGE IN IBD: MAPPING READINESS, MOBILE HEALTH PREFERENCES, AND ENGAGEMENT

**DOI:** 10.1093/jcag/gwaf042.126

**Published:** 2026-02-13

**Authors:** M Eisele, A Lewis, S Busse, B Toews, N Haskey, C Lu, H Singh, B Oketola, M Yousuf, L M Taylor, M Raman

**Affiliations:** Internal Medicine, University of Calgary Cumming School of Medicine, Calgary, AB, Canada; Cumming School of Medicine, University of Calgary, Calgary, AB, Canada; Gastroenterology, University of Calgary Cumming School of Medicine, Calgary, AB, Canada; Internal Medicine, University of Calgary Cumming School of Medicine, Calgary, AB, Canada; Biology, The University of British Columbia, Vancouver, BC, Canada; Medicine, University of Calgary, Calgary, AB, Canada; University of Manitoba Max Rady College of Medicine, Winnipeg, MB, Canada; University of Manitoba Max Rady College of Medicine, Winnipeg, MB, Canada; Internal Medicine, University of Calgary Cumming School of Medicine, Calgary, AB, Canada; Cumming School of Medicine, University of Calgary, Calgary, AB, Canada; Internal Medicine, University of Calgary Cumming School of Medicine, Calgary, AB, Canada

## Abstract

**Background:**

A Mediterranean diet (MD), regular moderate to vigorous physical activity (MVPA), and adequate sleep have demonstrated beneficial effects on inflammatory pathways in inflammatory bowel disease (IBD). Yet, patients with IBD struggle to meet these targets. Behaviour change (BC) is complex and nuanced, requiring different levels of support depending on the individual’s BC stage. Mobile health (mHealth) tools offer a practical way to provide personalized, behavior-focused support.

**Aims:**

To characterize lifestyle behaviours, preferences of behaviour change support, and stages of BC among IBD patients.

**Methods:**

A prospective survey assessing lifestyle behaviours and preferences for mHealth tools guided by Michie et al.’s BC Taxonomy was administered to IBD patients during routine clinic visits. Participants who completed the survey were offered LyfeMD, a holistic mHealth app supporting diet, physical activity, sleep, and mindfulness. App use was monitored and participants were classified according to the Transtheoretical model of BC stages: pre-contemplation (no interest in BC), contemplation (interest in LyfeMD but no action), preparation (downloaded LyfeMD but no engagement), action (active LyfeMD use), and maintenance (sustained use for ≥3 months).

**Results:**

Of the 138 IBD patients recruited, 90.6% completed the survey of which 56.5% were female and 70.4% in remission. Diet quality was poor, with 37.0% showing risk of malnutrition and low adherence to the MD, particularly deficient in fish, olive oil, and vegetables. Only 29.7% met physical activity recommendations (≥150 MVPA/week), and 75.6% reported no vigorous activity (Table 1). Additionally, 51.1% reported poor sleep, averaging 6.8 hours per night (Table 1). Regarding BC readiness, 24.8% were in pre-contemplation, 37.6% in contemplation, 4.0% in preparation, and 33.6% in action, therefore in total 94 (75.2%) participants showed interest in LyfeMD. Among those with sufficient follow-up time, 6 of 17 (35.3%) maintained LyfeMD use beyond three months. Figur 1. shows participants’ preferences in a mHealth tool.

**Conclusions:**

Lifestyle behaviors remain suboptimal among people with IBD, emphasizing the need for strategies to support individualized behavior change. While interest in mHealth tools was high, demonstration of progression from contemplation to action was limited. Future mHealth tools should emphasize evidence-based education materials specifically on diet, include tracking tools, and provide additional support to facilitate contemplative IBD patients toward action.

**Funding Agencies:**

None